# Status and Trends of the Association Between Diabetic Nephropathy and Diabetic Retinopathy From 2000 to 2021: Bibliometric and Visual Analysis

**DOI:** 10.3389/fphar.2022.937759

**Published:** 2022-06-20

**Authors:** Wenwen Lin, Yayong Luo, Fang Liu, Hangtian Li, Qian Wang, Zheyi Dong, Xiangmei Chen

**Affiliations:** ^1^ School of Clinical Medicine, Guangdong Pharmaceutical University, Guangzhou, China; ^2^ National Clinical Research Center for Kidney Diseases, State Key Laboratory of Kidney Diseases, Beijing Key Laboratory of Kidney Disease Research, First Medical Center of Chinese PLA General Hospital, Nephrology Institute of the Chinese People’s Liberation Army, Beijing, China

**Keywords:** diabetic nephropathy, diabetic retinopathy, diabetes, bibliometrics, citespace

## Abstract

**Background:** Diabetic nephropathy (DN) and diabetic retinopathy (DR) are microvascular complications of diabetes that share a similar pathogenesis and clinical relevance. The study aimed to visually analyze the research status and development trend of the relationship between DN and DR by means of bibliometrics and knowledge mapping.

**Methods:** Publications were collected from the Science Citation Index-Expanded of the Web of Science Core Collection between 2000 and 2021. CiteSpace, Alluvial Generator, and Microsoft Excel were used to analyze and present the data.

**Results:** A total of 3,348 publications were retrieved and 3,285 were included in the analysis after deduplication. The publications demonstrated an annually increasing trend. The results of the collaborative network analysis showed that the United States, Steno Diabetes Center, and Tien Y. Wong were the most influential country, institution and author, in this field of research, respectively. The analysis of references and keywords showed that the pathogenesis of DN and DR and their relationship with cardiovascular disease are research hotspots. The clinical relevance and drug therapy for DN and DR will become frontiers of future research in this field.

**Conclusion:** This study is the first to visualize the correlation between DN and DR using a bibliometric approach. This study provides a reference of research trends for scholars.

## Introduction

Diabetes is a worldwide public health problem that affects millions of people of all ages, sexes, and racial and ethnic groups (Rangel et al., 2019). The International Diabetes Federation (IDF) report estimated that in 2021, 536.6 million people worldwide had diabetes. These figures are expected to increase to 783.2 million by 2045 (Sun et al., 2022). Diabetic nephropathy (DN) and diabetic retinopathy (DR) are two major microvascular complications of diabetes that increase all-cause and cardiovascular disease (CVD)-related mortality (Sabanayagam et al., 2019).

DN is the leading cause of end-stage renal disease, accounting for approximately 50% of cases in developed countries (Tuttle et al., 2014). DN is a clinical syndrome characterized by persistent albuminuria, elevated arterial blood pressure, a relentless decline in the glomerular filtration rate, and a high risk of cardiovascular morbidity and mortality (Jawa et al., 2004). Moreover, the prevalence rate of DN in patients with type 2 diabetes is reported to be 41.3% (Tong et al., 2020).

DR is the most common and serious ocular complication of diabetes (Cheung et al., 2010). It is the leading cause of blindness and visual impairment in adults (Congdon et al., 2003). More than one-third of people with diabetes have signs of DR, and one-third of those with DR have vision-threatening DR (Yau et al., 2012). DN and DR share a similar pathogenesis and common risk factors (Tsilibary, 2003; Wong et al., 2014). Srivastav et al. revealed that in DR, deranged serum urea and creatinine levels can augment the damage to the retinal neural tissue (Srivastav et al., 2015). Additionally, Zhuang et al. demonstrated that stage of estimated glomerular filtration rate (eGFR) and urine albumin-to-creatinine ratio (UACR) were associated with the stage of DR and diabetic macular edema development (Zhuang et al., 2019). Finally, vascular abnormalities in DR are predictors of impaired renal function and microalbuminuria is an accurate biomarker of DR progression (Pan et al., 2021).

Currently, renal microvessel examination is mainly performed by histological examination, namely renal biopsy, which is an invasive operation. The retinal microvasculature is the only part of the human circulatory system that can be directly visualized non-invasively *in vivo*, readily photographed, and subjected to digital image analysis (Patton et al., 2006). Thus, the retinal vessels offer a unique and easily accessible window to study the health and disease of the human microcirculation. Moreover, the kidney and retina have similar anatomical and physiological characteristics (Cheung et al., 2012). Therefore, the study of the correlation between DN and DR will provide the possibility of non-invasive operation for the detection of DN.

Bibliometrics is seen as a valuable quantitative and qualitative method for evaluating scientific production, and its influence is growing (Ellegaard and Wallin, 2015; Liu et al., 2021). Bibliometrics applies mathematical and statistical methods to the analysis of scholarly publications to understand research hotspots and research fronts in specific fields of study. Many current applications of bibliometrics in medical science and healthcare can be used to discover new information about academic trends, pharmacotherapy, disease, and broader health sciences trends (Thompson and Walker, 2015). The results of bibliometric analysis help to identify gaps in research hotspots and can be used for future studies with more forward connections (Kumar et al., 2021; Kumar and Goel, 2021). Bibliometrics has been applied to the respective fields of DN and DR (Zou and Sun, 2019; Li X. J. et al., 2021), and previous studies have demonstrated a link between these two diseases (Yun et al., 2016; Hsieh and Hsieh, 2021). However, no systematic visual analysis of topics in this field has been performed using bibliometric methods. Therefore, this study aimed to visually analyze the research status and development trends of the relationship between DN and DR by means of bibliometrics and knowledge mapping.

## Materials and Methods

**FIGURE 1:**
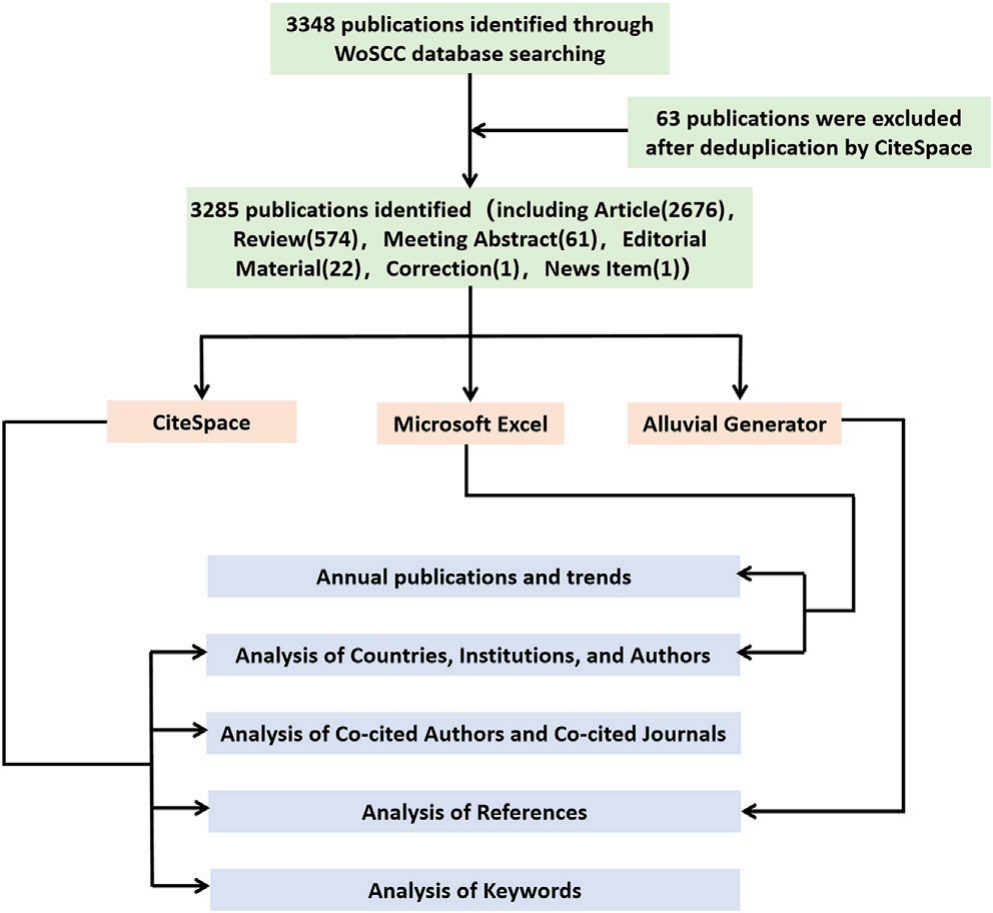
Flow diagram of the publications screening process and bibliometric analysis methods.

To analyze research trends, references and keywords analysis of the association between DN and DR, CiteSpace, Alluvial Generator, and Microsoft Excel are used (Figure 1).

### Data Source and Search Strategy

Science Citation Index Expanded (SCI-E) from the Web of Science Core Collection (WoSCC) was used as the data source. The search expressions were constructed as follows: TS = (Diabetic Retinopathy) AND TS = (Diabetic Nephropathies or Diabetic Nephropathy or Diabetic Kidney Disease or Diabetic Glomerulosclerosis or Intracapillary Glomerulosclerosis or Nodular Glomerulosclerosis or Kimmelstiel-Wilson Syndrome or Kimmelstiel Wilson Syndrome or Kimmelstiel-Wilson Disease or Kimmelstiel Wilson Disease). The publication period in this study was set between 2000 and 2021. All searches were completed on the same day on February 24, 2022. No restrictions on the type or language of the publications was set. A total of 3,348 publications were retrieved, and after deduplication with CiteSpace, 3,285 publications were included in the analysis (Figure 1). These 3,285 publications were exported to CiteSpace and Alluvial Generator for further analysis.

### Bibliometric Analysis and Software Assistance

In this study, CiteSpace v.5.8. R3 (64-bit) software was used to draw a knowledge map of the research status and trends of the association between DN and DR. CiteSpace is an information visualization software developed by Professor Chen Chaomei using Java language. CiteSpace supports visual exploration with knowledge discovery in bibliographic databases. It provides a wide range of users with a visual mapping tool to explore areas of expertise and the emergence of research topics within knowledge areas, and to identify emerging trends and transient patterns in the scientific literature (Chen and Chen, 2005; Synnestvedt et al., 2005).

The visual graphs produced by CiteSpace consist of nodes and lines. The size of the node represents the frequency. Moreover, the connection between nodes represents the strength of cooperation; the thicker the connection, the stronger the cooperation. In addition, nodes with betweenness centrality >0.1 are usually marked with purple circles. The larger the centrality value, the more cooperative the node is with other nodes. Log-Likelihood Ratio (LLR) algorithm was chosen to cluster co-cited references and keywords. Each cluster is composed of multiple closely related words. The smaller the number of cluster labels, the more references or keywords are included in that cluster. The silhouette (S) value refers to the average contour value of the cluster. It is generally considered that, with an S > 0.5, the cluster is reasonable, while with an S > 0.7, the cluster is convincing (Chen et al., 2012).

For this study, the specific parameters of CiteSpace were set as follows: 1) Time Slicing: From January 2000 to December 2021; Years Per Slice: 1; 2) Term Source: Title, Abstract, Author, Keywords, and Keyword Plus; 3) Node Types: Author, Institution, Country, Keyword, Reference, Cited Author, and Cited Journal; 4) Selection Criteria: Top N = 50.

Alluvial Generator was used to draw an alluvial flow map showing the change process of co-cited documents in the past 5 years (2017-2021). The modules of publications cited in these five consecutive years are colored, indicating that the document has received a high degree of attention in this time frame.

Microsoft Excel was used to construct tables, make rose charts, and demonstrate the annual national trends in publications.

## Results

There were 6 document types in 3,285 publications from 2000 to 2021. Among them, articles were the most (2,676), followed by reviews (574), meeting abstracts (61), editorial materials (22), collection 1) and news item 1) (Figure 1).

### Annual Publications and Trends

**FIGURE 2:**
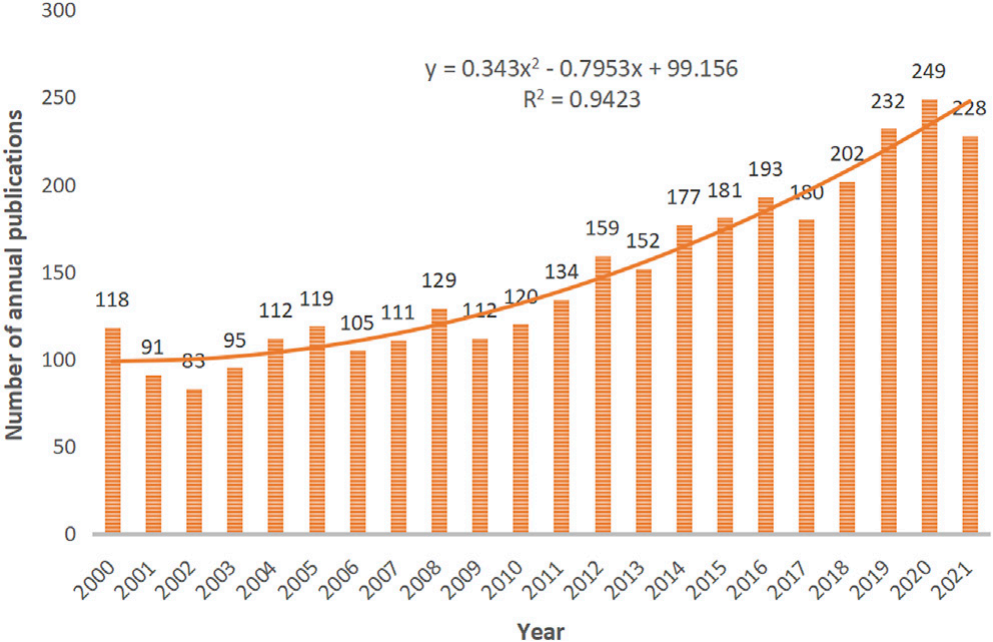
The number of annual publications on DN and DR research between 2000 and 2021. The horizontal coordinates represent the year of publication. The vertical coordinates represent the number of publications.

From 2000 to 2021, the SCI-E of the WoSCC database included a total of 3,285 publications on DN and DR. As shown in Figure 2, the number of publications related to DN and DR from 2000 to 2021 showed a fluctuating upward trend. The number of publications in 2001, 2002, 2006, 2009, 2013, 2017, and 2021 declined, while the number of publications in the rest of the years rose. The most publications in 1 year occurred in 2020 and reached 249 articles. The polynomial curve fitting between year and number of annual publications was drawn to better understand the trends in publications on DN and DR. As presented in Figure 2, the correlation coefficient R-square of the equation reached 0.9423.

### Analysis of Countries, Institutions, and Authors

**FIGURE 3:**
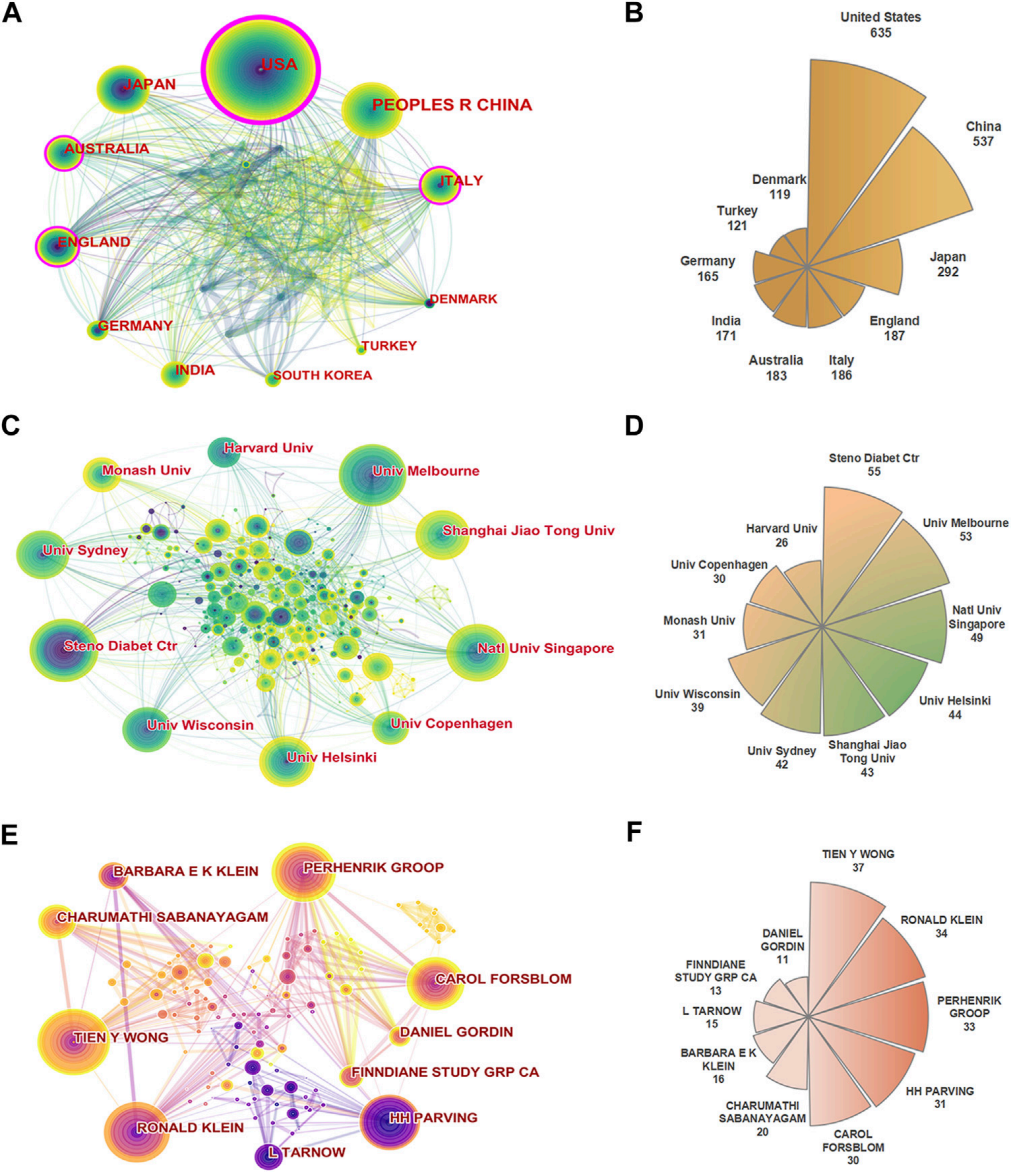
**(A)** The collaboration network of countries. **(B)** Rose chart of the top 10 productive countries. **(C)** The collaboration network of institutions. **(D)** Rose chart of the top 10 productive institutions. **(E)** The collaboration network of authors. **(F)** Rose chart of the top 10 productive authors.

To understand the contribution of each country to the research field of DN and DR, the global collaboration network analysis is shown in Figure 3A. The United States (635) had the most publications, followed by China (537), Japan (292), England (187), and Italy (186) (Figure 3B). Regarding the centrality of countries, the United States (0.32) ranked first, followed by Italy (0.17), Australia (0.14), England (0.13), and Saudi Arabia (0.08) (Table 1). The United States is not only a major research center on DN and DR, but also has close cooperation with many countries such as China, Australia, and Japan in this field of research.

**TABLE 1:** Top 10 publication counts and centralities of countries, institutions, and authors.

Items	Rank	Count	Name	Rank	Centrality	Name
Country	1	635	United States	1	0.32	United States
	2	537	China	2	0.17	Italy
	3	292	Japan	3	0.14	Australia
	4	187	England	4	0.13	England
	5	186	Italy	5	0.08	Saudi Arabia
	6	183	Australia	6	0.08	Malaysia
	7	171	India	7	0.07	Germany
	8	165	Germany	8	0.06	India
	9	121	Turkey	9	0.06	France
	10	119	Denmark	10	0.05	China
Institution	1	55	Steno Diabet Ctr	1	0.09	Steno Diabet Ctr
	2	53	Univ Melbourne	2	0.09	Harvard Univ
	3	49	Natl Univ Singapore	3	0.09	Univ Tokyo
	4	44	Univ Helsinki	4	0.08	Univ Melbourne
	5	43	Shanghai Jiao Tong Univ	5	0.08	Natl Univ Singapore
	6	42	Univ Sydney	6	0.06	Univ Toronto
	7	39	Univ Wisconsin	7	0.05	Univ Helsinki
	8	31	Monash Univ	8	0.05	Shanghai Jiao Tong Univ
	9	30	Univ Copenhagen	9	0.05	Tianjin Med Univ
	10	26	Harvard Univ	10	0.04	Univ Sydney
Author	1	37	Tien Y. Wong	1	0.01	Tien Y. Wong
	2	34	Ronald Klein	2	0.01	Ronald Klein
	3	33	Per-henrik Groop	3	0.01	Per-henrik Groop
	4	31	Hans-Henrik Parving	4	0.01	Hans-Henrik Parving
	5	30	Carol Forsblom	5	0	Carol Forsblom
	6	20	Charumathi Sabanayagam	6	0	Charumathi Sabanayagam
	7	16	Barbara E. K. Klein	7	0	Barbara E. K. Klein
	8	15	Lise Tarnow	8	0	Lise Tarnow
	9	13	FinnDiane Study Group	9	0	FinnDiane Study Group
	10	11	Daniel Gordin	10	0	Daniel Gordin

Through the analysis of research institutions, scholars can understand the global distribution of institutions conducting research on the correlation between DN and DR, and then provide a basis for seeking cooperating institutions during research (Figure 3C). The top 10 institutions according to the number of publications came from 5 countries, namely Denmark (Steno Diabetes Center, University of Copenhagen), Australia (University of Melbourne, University of Sydney, Monash University), Singapore (National University of Singapore), Finland (University of Helsinki), China (Shanghai Jiao Tong University), and the United States (University of Wisconsin, Harvard University). The institution with the most publications was Steno Diabetes Center (55), followed by University of Melbourne (53), National University of Singapore (49), University of Helsinki (44) and Shanghai Jiao Tong University (43). In terms of centrality, the top 5 institutions were Steno Diabetes Center (0.09), Harvard University (0.09), University of Tokyo (0.09), University of Melbourne (0.08) and National University of Singapore (0.08) (Figure 3D; Table 1).

Figure 3E shows the collaboration network of authors, which provides a basis for finding research partners and identifying industry giants. The author with the most publications was Tien Y. Wong (37), followed by Ronald Klein (34), Per-henrik Groop (33), Hans-Henrik Parving (31), and Carol Forsblom (30) (Figure 3F). The top 4 authors according to centrality were Tien Y. Wong (0.04), Ronald Klein (0.04), Per-henrik Groop (0.04), and Hans-Henrik Parving (0.04) (Table 1).

### Analysis of Co-Cited Authors and Co-Cited Journals

**FIGURE 4:**
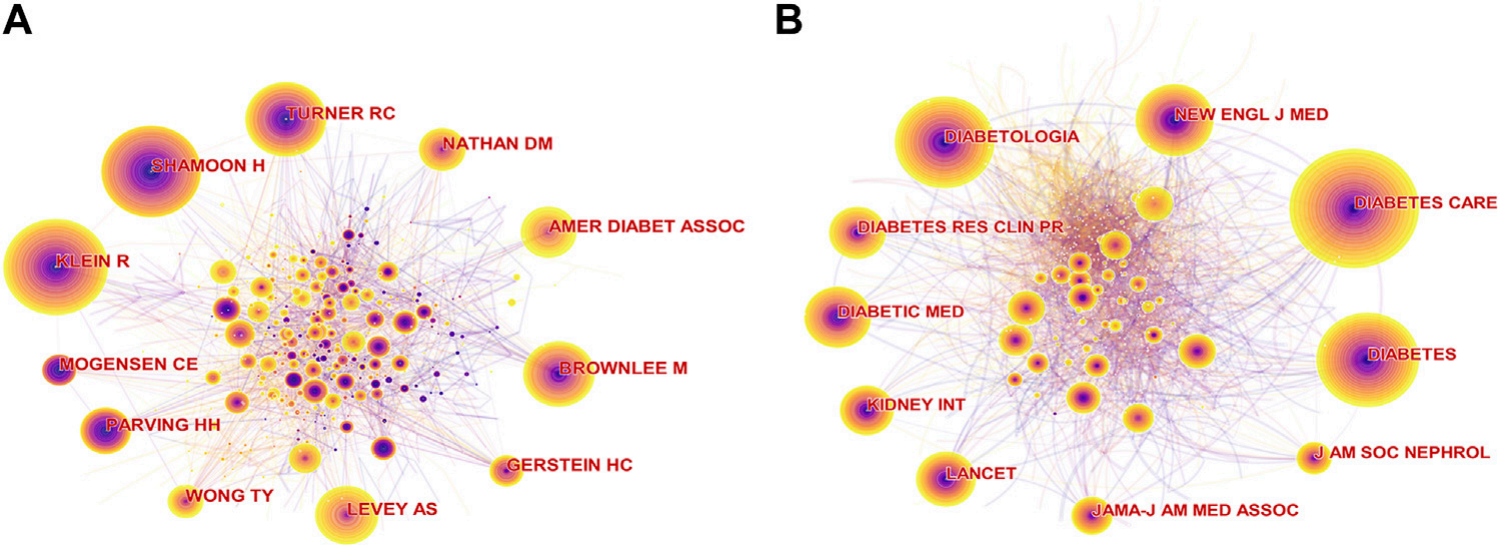
**(A)** The collaboration network of co-cited authors. **(B)** The collaboration network of co-cited journals.

Co-cited authors in this field of research are presented in Figure 4A. The top 5 co-cited authors by citation frequency were Ronald Klein (486), Harry Shamoon (471), Robert C. Turner (388), Michael Brownlee (347), and Andrew S. Levey (308). The top 5 co-cited authors for centrality were Lloyd Paul Aiello (0.08), followed by Michael Brownlee (0.07), Hans-Henrik Parving (0.07), Paola Fioretto (0.07), and Antonio Ceriello (0.06) (Table 2).

**TABLE 2:** Top 10 publication counts and centralities of co-cited authors and co-cited journals.

Items	Rank	Count	Name	Rank	Centrality	Name
Co-cited author	1	486	Ronald Klein	1	0.08	Lloyd Paul Aiello
	2	471	Harry Shamoon	2	0.07	Michael Brownlee
	3	388	Robert C. Turner	3	0.07	Hans-Henrik Parving
	4	347	Michael Brownlee	4	0.07	Paola Fioretto
	5	308	Andrew S. Levey	5	0.06	Antonio Ceriello
	6	274	American Diabetes Association	6	0.05	Robert C. Turner
	7	264	Hans-Henrik Parving	7	0.05	Andrzej S. Krolewski
	8	240	David M. Nathan	8	0.05	Hans-Peter Hammes
	9	192	Carl Erik Mogensen	9	0.05	Nish Chaturvedi
	10	188	Hertzel C. Gerstein	10	0.05	Trevor J. Orchard
Co-cited journal	1	2,517	Diabetes Care	1	0.05	J. Cardiovasc. Pharm.
	2	2,102	Diabetologia	2	0.04	Eur. J. Pharmacol.
	3	2018	Diabetes	3	0.04	Curr. Eye Res.
	4	1698	New Engl. J. Med.	4	0.04	Mol. Cell Biochem.
	5	1393	Diabetic Med.	5	0.04	Cancer Res.
	6	1321	Lancet	6	0.03	J. Intern. Med.
	7	1260	Diabetes Res. Clin. Pr.	7	0.03	Free Radical Bio. Med.
	8	1246	Kidney Int.	8	0.03	Endocrinology
	9	1043	JAMA	9	0.03	Febs. Lett.
	10	932	J. Am. Soc. Nephrol.	10	0.03	Brit. J. Pharmacol.

In the analysis of co-cited journals (Figure 4B), identifying the core journals in the field is beneficial. The highest frequency among co-cited journals was Diabetes Care (2,517), followed by Diabetologia (2,102), Diabetes (2018), New Engl. J Med. (1698), and Diabetic Med. (1393). The top 5 co-cited journals for centrality were J. Cardiovasc. Pharm. (0.05), Eur. J. Pharmacol. (0.04), Curr. Eye Res. (0.04), Mol. Cell Biochem. (0.04), and Cancer Res. (0.04) (Table 2).

### Analysis of References

**FIGURE 5:**
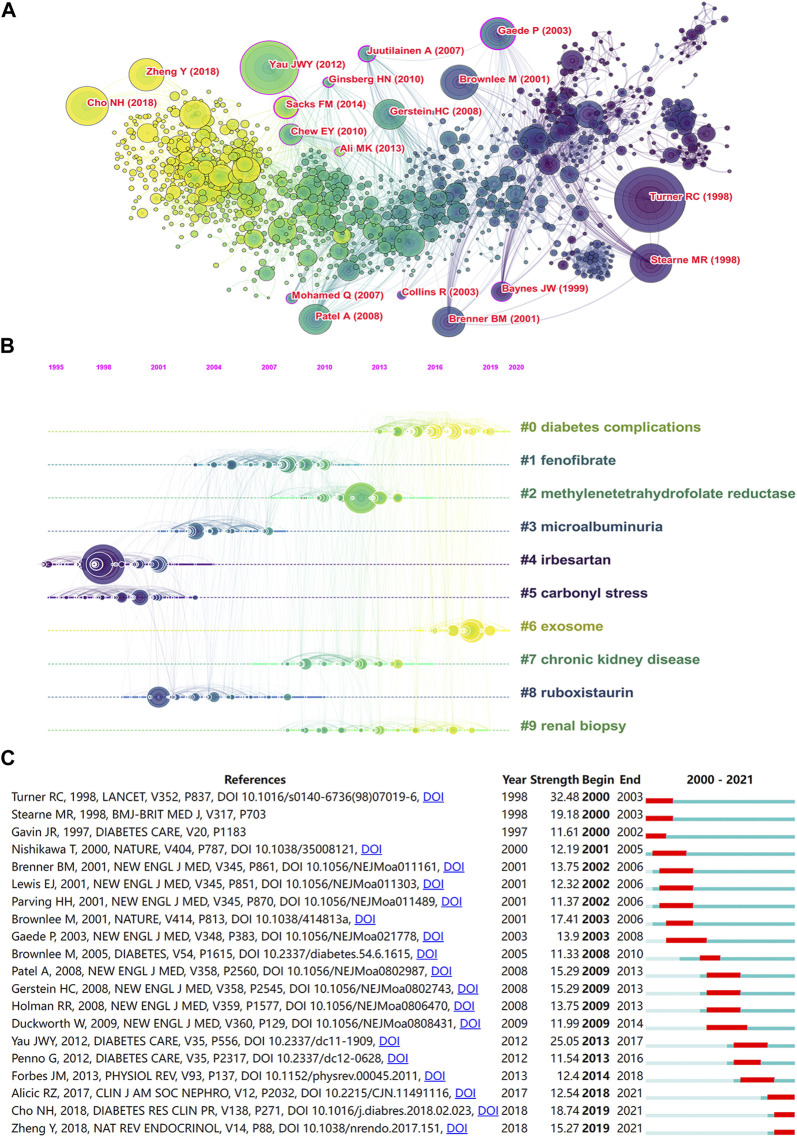
**(A)** The network of co-cited references. **(B)** The timeline view network of co-cited references. **(C)** The top 20 references with the strongest citation bursts.

**FIGURE 6:**
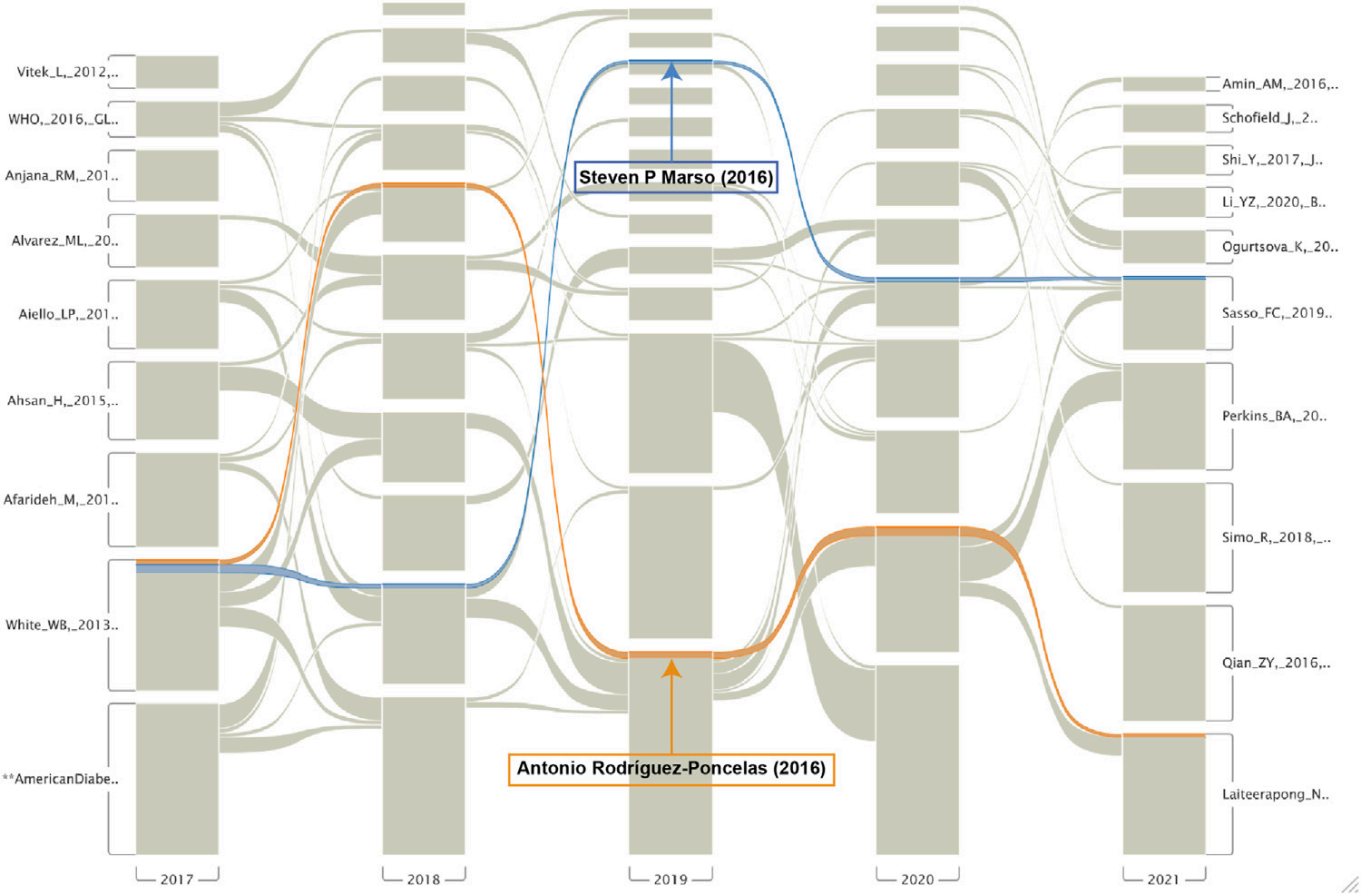
The alluvial flow map of co-cited references from 2017 to 2021.

As shown in Figure 5A and Table 3, “Turner RC (1998), Yau et al. (2012), Stearne MR (1998), and Cho NH (2018)” were frequently cited references. Top 10 clusters of co-cited references were found. The S values of the top 10 clusters were all above 0.7, indicating that these clusters were convincing (Table 4). The top five cluster labels were “diabetes complications,” “fenofibrate,” “methylenetetrahydrofolate reductase,” “microalbuminuria,” and “irbesartan.” The largest cluster of “diabetes complications” indicated that studies related to diabetes complications may be the current research hotspot. The timeline view of co-cited references (Figure 5B) shows that irbesartan and carbonyl stress were early research areas for DN and DR. Additionally, diabetes complications and exosome were shown to be the current research hotspots in DN and DR. The top 20 references with the strongest citation bursts are shown in Figure 5C. The highest burst strength was from Turner RC (1998). Furthermore, Radica Z. Alicic (2017), NH Cho (2018), and Yan Zheng (2018) received more attention in recent years. The alluvial flow map shown in Figure 6 represents the most cited references from 2017 to 2021, with Marso et al. (2016) and Rodríguez-Poncelas (2016) cited for five consecutive years.

**TABLE 3:** Top 10 co-cited references.

Rank	Frequency	Title	Author	Year	Journal
1	64	Intensive blood-glucose control with sulphonylureas or insulin compared with conventional treatment and risk of complications in patients with type 2 diabetes (UKPDS 33)	Robert C. Turner	1998	Lancet
2	52	Global Prevalence and Major Risk Factors of Diabetic Retinopathy	Joanne W.Y. Yau	2012	Diabetes Care
3	38	Tight blood pressure control and risk of macrovascular and microvascular complications in type 2 diabetes: UKPDS 38	M. R. Stearne	1998	BMJ
4	38	IDF Diabetes Atlas: Global estimates of diabetes prevalence for 2017 and projections for 2045	N.H. Cho	2018	Diabetes Res. Clin. Pr.
5	34	Biochemistry and molecular cell biology of diabetic complications	Michael Brownlee	2001	Nature
6	31	Multifactorial Intervention and Cardiovascular Disease in patients with Type 2 Diabetes	Peter Gaede	2003	New Engl. J. Med.
7	31	Global aetiology and epidemiology of type 2 diabetes mellitus and its complications	Yan Zheng	2018	Nat. Rev. Endocrinol.
8	30	Intensive Blood Glucose Control and Vascular Outcomes in Patients with Type 2 Diabetes	Anushka Patel	2008	New Engl. J. Med.
9	30	Effects of Intensive Glucose Lowering in Type 2 Diabetes	H.C. Gerstein	2008	New Engl. J. Med.
10	29	Effects of losartan on renal and cardiovascular outcomes in patients with type 2 diabetes and nephropathy	Barry M. Brenner	2001	New Engl. J. Med.

**TABLE 4:** Top 10 largest clusters of co-cited references.

Cluster ID	Size	Silhouette	Mean year	Top terms (LLR)
#0	185	0.809	2015	diabetes complications
#1	153	0.857	2007	fenofibrate
#2	118	0.872	2011	methylenetetrahydrofolate reductase
#3	109	0.89	2004	microalbuminuria
#4	96	0.92	1998	irbesartan
#5	93	0.954	1999	carbonyl stress
#6	83	0.937	2017	exosome
#7	81	0.917	2010	chronic kidney disease
#8	73	0.928	2003	ruboxistaurin
#9	62	0.95	2014	renal biopsy

### Analysis of Keywords

**FIGURE 7:**
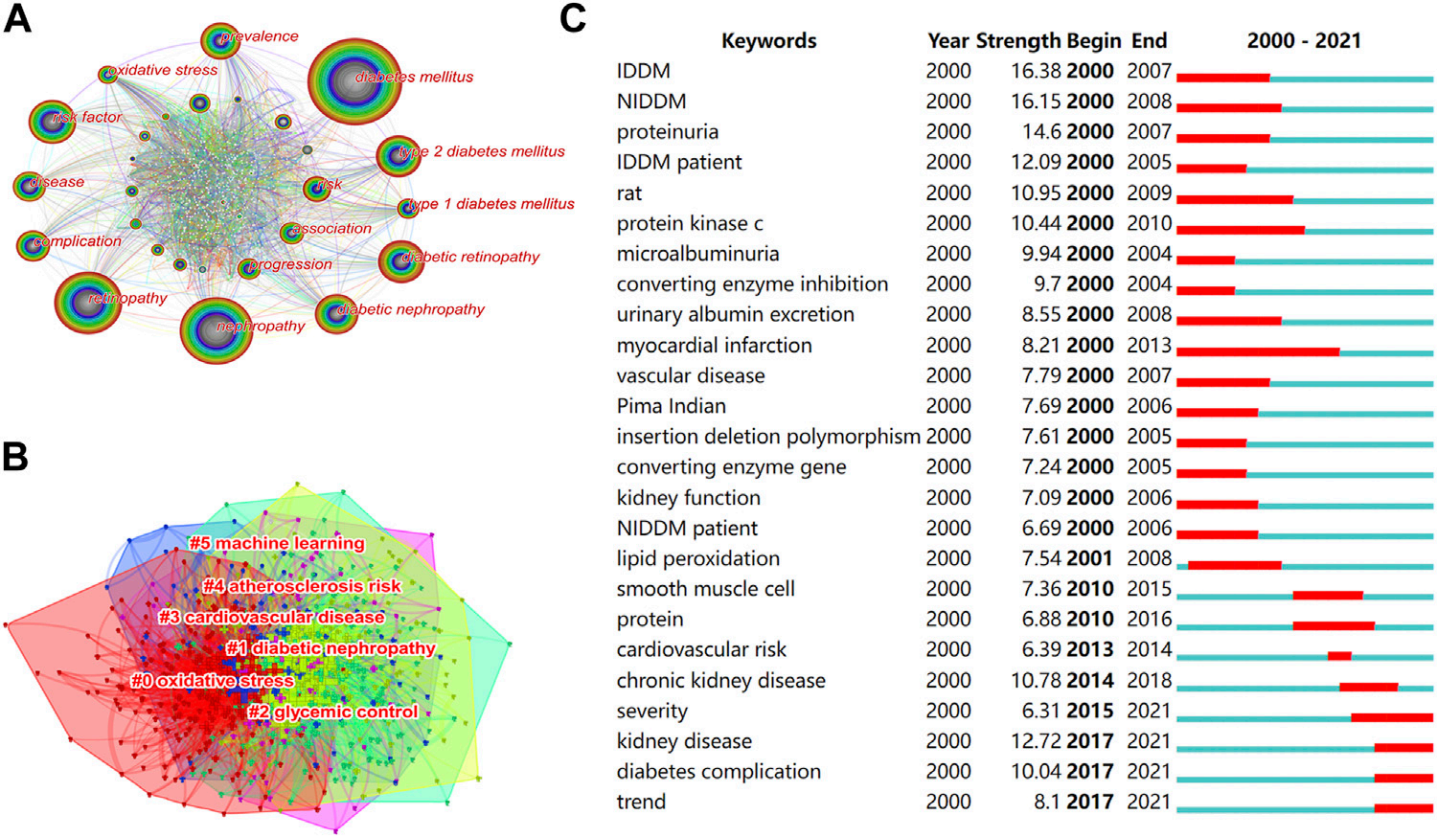
**(A)** The co-occurrence network of keywords. **(B)** The clusters of keywords. **(C)** The top 25 keywords with the strongest citation bursts.

Research topics with high-frequency keyword performance were considered research hotspots in the field. “Diabetes mellitus,” “nephropathy,” “retinopathy,” “risk factor,” “diabetic retinopathy,” “type 2 diabetes mellitus,” “diabetic nephropathy,” “prevalence,” “disease,” and “complication” were high-frequency keywords (Figure 7A). The top 10 keywords for centrality were “glycation end product,” “diagnosis,” “neuropathy,” “insulin,” “follow up,” “diabetic nephropathy,” “oxidative stress,” insulin resistance,” “glomerular filtration rate,” and “coronary heart disease” (Table 5). Figure 7B shows the cluster analysis of keywords. The S values of the top six largest clusters were all >0.5, indicating that the clustering network was reasonable. The top six largest cluster labels were “oxidative stress,” “diabetic nephropathy,” “glycemic control,” “cardiovascular disease,” “atherosclerosis risk,” and “machine learning” (Table 6). Burst keywords can judge the change of research trends and intuitively show the research hotspots in recent years. Top 25 keywords with the strongest citation bursts are shown in Figure 7C. The term with the highest burst strength was “IDDM”. Moreover, “severity,” “kidney disease,” “diabetes complication,” and “trend” were the most recent keywords that appeared in the last 6 years.

**TABLE 5:** Top 10 keywords by frequency and centrality.

Rank	Count	Keyword	Centrality	Keyword
1	1005	diabetes mellitus	0.05	glycation end product
2	785	nephropathy	0.05	diagnosis
3	727	retinopathy	0.05	neuropathy
4	552	risk factor	0.05	insulin
5	549	diabetic retinopathy	0.05	follow up
6	526	type 2 diabetes mellitus	0.04	diabetic nephropathy
7	507	diabetic nephropathy	0.04	oxidative stress
8	493	prevalence	0.04	insulin resistance
9	413	disease	0.04	glomerular filtration rate
10	410	complication	0.04	coronary heart disease

**TABLE 6:** Top 6 largest clusters of keywords.

Cluster ID	Silhouette	Mean year	Top terms (LLR)
0	0.674	2007	oxidative stress
1	0.718	2005	diabetic nephropathy
2	0.59	2010	glycemic control
3	0.672	2009	cardiovascular disease
4	0.748	2008	atherosclerosis risk
5	0.98	2020	machine learning

## Discussion

### General Information

Publications regarding the association between DN and DR showed an overall upward trend between 2000 and 2021. The United States was the country with the highest number of publications in the field. Two of the top ten institutions in terms of publications were from the United States, namely the University of Wisconsin and Harvard University. The centrality of the United States was the highest, representing the close cooperation between the United States and other countries. The most frequently cited document (citations = 2,798) of the United States was published by Ferdinando Giacco and Michael Brownlee. It mainly described the progress made in understanding oxidative stress and its role in the development of diabetic complications (Giacco and Brownlee, 2010).

The institution with the most publications and greatest centrality ranking was Steno Diabetes Center from Denmark, with 55 publications covering the epidemiology, progression, risk factors, biomarkers, genetics, and other aspects of microvascular complications of diabetes (Rossing et al., 2002; Rossing et al., 2004; Rossing, 2005; Alkhalaf et al., 2010; Alkayyali and Lyssenko, 2014). The most cited of these was a cross-sectional study published by Hans-Henrik Parving evaluating 32,208 patients with type 2 diabetes from 33 countries without known proteinuria. The results of this study showed that retinopathy was an independent risk factor for microalbuminuria. The study also showed a high global prevalence of microalbuminuria and decreased renal function detected in patients with type 2 diabetes without known renal disease, both of which are associated with increased renal and cardiovascular risk (Parving et al., 2006). Additionally, the University of Melbourne from Australia was working on clinical research on the relationship between DN and DR. The institution found that changes in the retinal vascular caliber can predict the risk of DN (Wong et al., 2004; Klein et al., 2007). Thus, assessing the risk of DN by noninvasive testing of retinal microvessels in diabetic patients may be possible. Gilbert et al. found that angiotensin converting enzyme inhibition and angiotensin receptor blockade could reduce the development and progression of diabetic nephropathy, cardiovascular disease, and possibly retinopathy (Gilbert et al., 2003).

The results showed that Tien Y. Wong (University of Melbourne, National University of Singapore) had the most publications. His main focus was on the correlation of retinal blood vessels with chronic kidney disease, demonstrating that retinal microangiopathy is associated with the development of DN in diabetic patients (Klein et al., 2007; Broe et al., 2014; Yip et al., 2015; Nusinovici et al., 2021). Moreover, Diabetes Care was the most co-cited journal. One of the most cited publications in Diabetes Care addressed the role of oxidative stress in the development of diabetic complications (Giacco and Brownlee, 2010). The latest research of this journal has focused on sodium-glucose cotransporter 2 (SGLT2) inhibitors and DN. SGLT2 inhibitors are nephroprotective and play a role in the primary prevention of DN (Mosenzon et al., 2021; Nagasu et al., 2021). Matthews et al. found that SGLT2 inhibition may be a useful therapeutic approach to prevent the development of DR and lessen its severity if administered early in the disease process (Matthews et al., 2022).

### Hotspots and Frontiers

Cluster analysis of co-cited reference and keyword showed that the pathogenesis of DN and DR and their relationship with CVD were research hotspots. The largest cluster for co-cited reference was labeled as “diabetes complications.” The most relevant citer in the cluster was Winston Crasto. He has discussed the microvascular complications of diabetes, including DN, DR, and diabetic neuropathy, and provides best practice clinical care recommendations to guide health care professionals to better manage people with these conditions (Crasto et al., 2021). Additionally, cluster #2 was labeled as “methylenetetrahydrofolate reductase (MTHFR)”. The results of a study on the association of MTHFR polymorphisms with DR and DN in Japanese patients with type 2 diabetes suggest an important role of the MTHFR genotype in susceptibility to retinopathy under hyperglycemia, but not to nephropathy (Maeda et al., 2008). However, a meta-analysis of the MTHFR gene 677C/T polymorphism with DN and DR demonstrated that this genotype might confer a moderately augmented risk for DN and DR (Niu and Qi, 2012). The largest cluster of a keyword (#0) was that of “oxidative stress.” Oxidative stress, a cytopathic outcome of excessive generation of reactive oxygen species (ROS) and the repression of antioxidant defense system for ROS elimination, is involved in the pathogenesis of DN and DR (Jha et al., 2016; Kang and Yang, 2020). Cluster #2 was “glycemic control,” which is a significant risk factor for the development of diabetic complications (Nordwall et al., 2009). Hyperglycemia is an important indicator of risk for both DN with albuminuria or DR and less specific forms of CKD (Bash et al., 2008). Therefore, strict glycemic control can prevent diabetic microvascular complications, including DN and DR (Vasudevan et al., 2006). Cluster #3 was “cardiovascular disease”. A cross-sectional study showed that patients with type 1 diabetes and CKD without proliferative DR had a reduced prevalence of CVD (Gordin et al., 2018). Among people with diabetes, those with DN and DR had a higher risk of death from CVD (Sabanayagam et al., 2019). Continuing, cluster #4 was “atherosclerosis risk.” Atherosclerosis is the leading cause of heart disease and stroke (Lusis, 2000). Risk factors for atherosclerosis include hypertension, smoking, and diabetes mellitus (Libby et al., 2019). In patients with type 1 diabetes, atherosclerosis is associated with DR, and patients with DN have a greater coronary plaque burden than those with normoalbuminuria (Kim et al., 2007; Lovshin et al., 2018). Next, cluster #5 was “machine learning.” It has been found that artificial intelligence (AI)-based machine learning can predict microvascular complications in diabetic patients (Sambyal et al., 2021; Rashid et al., 2022).

A growing number of studies have demonstrated a correlation between DN and DR. Moriya et al. found that patients with type 2 diabetes with DR and concomitant microalbuminuria showed typical diabetic glomerulosclerosis and progressive renal dysfunction (Moriya et al., 2016). Previous studies have shown that in patients with type 2 diabetes, regardless of DR, microalbuminuria can cause early retinal microvascular changes (Cankurtaran et al., 2020; Ucgul et al., 2021). Thus, we may be able to identify early DN by detecting retinal microcirculation. Ansquer et al. has shown that fenofibrate offers an opportunity to prevent DN and DR (Ansquer et al., 2009). Furthermore, irbesartan is an angiotensin II receptor blocker that has become an important drug for the treatment of hypertension, heart failure, and prevention of DN (Michel et al., 2013). It has a protective effect on the kidneys in patients with type 2 diabetes and microalbuminuria (Parving et al., 2001). One study showed that angiotensin II receptor blockers play a potential role in the prevention and treatment of DR (Behl and Kotwani, 2017).

The top 10 co-cited references were related to the epidemiology of diabetes complications, drug interventions, molecular mechanisms, and CVD risk. The most frequently co-cited reference was a randomized controlled trial published by Robert C. Turner. The study suggested that intensive blood-glucose control by either sulphonylureas or insulin could substantially decrease the risk of microvascular complications in patients with type 2 diabetes (UK Prospective Diabetes Study Group, 1998a). The first milestone papers in this field of research were published in 1998. One reported that intensive blood-glucose control by either sulphonylureas or insulin substantially decreased the risk of microvascular complications (UK Prospective Diabetes Study Group, 1998a). This study is also the most frequently cited and has the most co-citations and highest burst strength. The other study reported that tight blood pressure control in diabetic patients delayed the progression of DR and renal failure (UK Prospective Diabetes Study Group, 1998b). Furthermore, the alluvial flow map showed two references that had been cited for five consecutive years from 2017 to 2021. Marso et al. found that liraglutide reduced the rate of first occurrence of death from cardiovascular causes, nonfatal myocardial infarction, or nonfatal stroke in patients with type 2 diabetes mellitus (Marso et al., 2016). Liraglutide appears to be effective in reducing proteinuria and improving renal function (Liu et al., 2019). In addition, liraglutide also has a certain protective effect on diabetic retinal injury (Wu et al., 2019). The team of Rodríguez-Poncelas showed that CKD, high UACR and low eGFR, appear to be associated with DR in patients with type 2 diabetes mellitus in Catalonia (Spain) (Rodriguez-Poncelas et al., 2016).

According to the analysis of burst keywords, research hotspots included the physiopathology, genetics, and biomarkers of DN and DR. We speculate that the study of the correlation between the severity of DN and DR will become a new trend. DN and DR have a similar pathogenesis, including oxidative stress (Jha et al., 2016; Kang and Yang, 2020), massive accumulation of glycation end products (Zong et al., 2011; Nishad et al., 2021), polyol pathway activation (Lewko et al., 2011), protein kinase C (Kamiya et al., 2003), and genetic factors (Alkayyali and Lyssenko, 2014). DN and DR share common clinical risk factors, including age, smoking, hypertension, obesity, and hyperlipidemia (Wong et al., 2014). Moreover, a study on the relationship between DN and DR in the Chinese population found that 48.8% of patients with type 2 diabetes mellitus diagnosed with DN by renal biopsy were accompanied by DR (Cao et al., 2019). Additionally, DR can be used to predict the risk and prognosis of DN (Zhang et al., 2018; Li Y. et al., 2021). It has also been found that DN is an independent risk factor for the development and progression of DR (Butt et al., 2020). Finally, the severity of DR is a risk factor for progression to CKD in patients with type 2 diabetes (Hsing et al., 2020).

### Limitations

This study is the first to visualize the correlation between DN and DR using a bibliometric approach; however, it still has some limitations. First, only the WOS core database was included in this study, and other databases, such as PubMed, Google Scholar, and Scope were not included. The primary data source for CiteSpace software is WOS (Synnestvedt et al., 2005). To meet the format requirements of the software, the WOS database is the primary choice. Other databases are not common in the practice of this software. Currently the software cannot automatically remove duplicates from different databases and analyze them simultaneously. Second, this study combined synonyms in the analysis, and the result bias due to subjectivity cannot be ruled out.

## Conclusion

This study systematically assessed the relationship between DN and DR based on a bibliometric analysis. The number of publications is annually increasing, and although a slight decline was observed in 2021, it is enough to show that this research field has continuously stimulated the interest of many scholars. Publications from different countries, institutions, authors, and journals were evaluated, showing their contributions to the field, which may also be used to guide future research. Through the analysis of references and keywords, we have predicted the future research hotspots and trends of DN and DR. For example, the pathogenesis of DN and DR and their relationship with CVD are current research hotspots. Moreover, the clinical relevance and drug therapy of DN and DR will be the frontiers of future research in this field. This study analyzes the status and trends of the relationship between DN and DR, which may promote the development of this research field. The study can provide scholars interested in this field with a reference of research trends and reduce their time to search for research hotspots and frontiers.

## Data Availability

The original contributions presented in the study are included in the article/Supplementary Material, further inquiries can be directed to the corresponding authors.
